# The *MDM2* Single-Nucleotide Polymorphism T309G Is Associated With the Development of Epimacular Membranes

**DOI:** 10.3389/fcell.2022.841660

**Published:** 2022-03-14

**Authors:** Heng Jiang, Bin Yan, Zhishang Meng, Lusi Zhang, Hetian Lei, Jing Luo

**Affiliations:** ^1^ Department of Ophthalmology, The Second Xiangya Hospital, Central South University, Changsha, China; ^2^ Shenzhen Eye Institute, Shenzhen Eye Hospital, Jinan University, Shenzhen, China

**Keywords:** *MDM2* SNP T309G, rs2279744, sanger sequencing, epimacular membrane, proliferative vitreoretinopathy, internal limiting membrane, macular hole

## Abstract

***Purpose:*** To investigate the role of the *mouse double minute 2* (*MDM2*) gene single-nucleotide polymorphism (SNP) T309G in the development of epimacular membranes (EMMs) by analyzing the genotype distribution and consistency of the polymorphism in paired membrane-blood samples.

***Methods:*** This was a cross–sectional genetic association study of patients with proliferative vitreoretinopathy (PVR) or EMMs. PVR membranes (PVRMs), internal limiting membranes (ILMs) (PVR-ILMs) and blood samples (PVR-blood) from patients with PVR, and EMMs, EMM-ILMs and EMM-blood from patients with EMMs were collected. The genotype of all samples was determined by Sanger sequencing. Sex composition, mean age, the genotype distribution of *MDM2* T309G, the allelic frequency of the *MDM2* SNP309 G allele (% G) and the somatic mutation rate at the *MDM2* T309G locus (% M) were analyzed and compared. The PVR and healthy Chinese donor groups were used as controls for different comparisons.

***Results:*** The EMM group of 62 patients was older than the PVR group of 61 patients by an average of 8.87 years (*p* < 0.0001), but the two groups were statistically similar in the sex composition (*p* = 0.1754). Importantly, G allele carriers were at a higher risk of developing EMMs than non-G allele carriers (*p* = 0.0479; OR = 2.047). Moreover, EMM-blood exhibited a significantly higher % G than blood samples from healthy Chinese donors (EMM-blood: 56.78%, donors: 45.61%; *p* = 0.0256; OR = 1.567). Regarding membrane-blood consistency, % M was significantly different between PVRMs and EMMs (PVRMs: 2.63%, EMMs: 21.57%; *p* = 0.0097; OR = 10.18) but not between different types of ILMs (PVR-ILMs: 18.18%, EMM-ILMs: 29.17%; *p* = 0.6855). Furthermore, EMMs (*p* = 0.0053; OR = 8.250) and EMM-ILMs (*p* = 0.0233; OR = 14.40) from patients with preoperative macular holes were more predisposed toward somatic mutations at the *MDM2* T309G locus than those from patients without preoperative macular holes.

***Conclusions:*** *MDM2* T309G is associated with the development of EMMs. Herein, the *MDM2* SNP309 G allele is first reported as an associated factor of EMMs in a Chinese population. In addition, EMMs and ILMs are genetically unstable at the *MDM2* T309G locus, especially when complicated with preoperative macular holes.

## Introduction

The tumor suppressor p53 is a pivotal component of cells that monitors and controls the most important cellular events, including cell cycle arrest, apoptosis, senescence, metabolism, DNA repair and malignant transformation. p53 acts as a switch between life (cell cycle arrest and attendant DNA repair) and death (apoptosis) and thus determines the fate of cells in response to cellular stress (Carvajal and Manfredi, 2013). The mouse double minute 2 (MDM2) homolog oncoprotein, an E3 ubiquitin ligase encoded by the proto-oncogene *MDM2*, is the primary negative regulator of p53. MDM2 represses p53-dependent transcription via a triple mechanism: nuclear or cytoplasmic ubiquitin-dependent proteasomal degradation of p53, reversible cytoplasmic p53 translocation and direct abrogation of p53-dependent transactivation. MDM2 deficiency leads to p53-driven embryonic lethality, which can be fully rescued by simultaneous deletion of p53 (Jones et al., 1995). In contrast, overexpression of MDM2 impairs p53-mediated regulation of cell growth, with the substantial accumulation of DNA damage, which supports tumorigenesis. Wade et al. (2013) reported that amplification of the *MDM2* gene or altered expression of the MDM2 protein is a feature of many tumors. In general, the expression of MDM2 is dependent on two promoters: the upstream P1 promoter in the first exon of the *MDM2* gene, which controls basal constitutive expression in normal living cells, and the downstream P2 promoter in the first intron, which initiates inducible expression in response to stimuli such as p53, RAS and estrogen receptor α under stress conditions (Barak et al., 1994). Transcripts derived from the P2 promoter exhibit enhanced translational efficiency that is approximately eight times that of transcripts derived from the P1 promoter (Landers et al., 1997). A naturally occurring single-nucleotide polymorphism (SNP) in the P2 promoter, *MDM2* T309G (rs2279744, a thymine-to-guanine change at the 309th nucleotide in the first intron), enhances binding of the transcriptional activator Sp1 to the P2 promoter and stimulates P2-driven inducible expression of MDM2 under stress, with subsequent attenuation of p53 signaling (Bond et al., 2004). Several clinical investigations have indicated that *MDM2* T309G is related to increased risk, early onset, high malignancy and poor prognosis in cancers (Boersma et al., 2006; Bougeard et al., 2006; Lind et al., 2006; Ohmiya et al., 2006; Gryshchenko et al., 2008). Moreover, Post et al. (2010) corroborated the correlation between *MDM2* T309G and increased tumor risk using two cohorts of genetically engineered mice carrying either the humanized *MDM2* SNP309 G allele or T allele.

**FIGURE 1:**
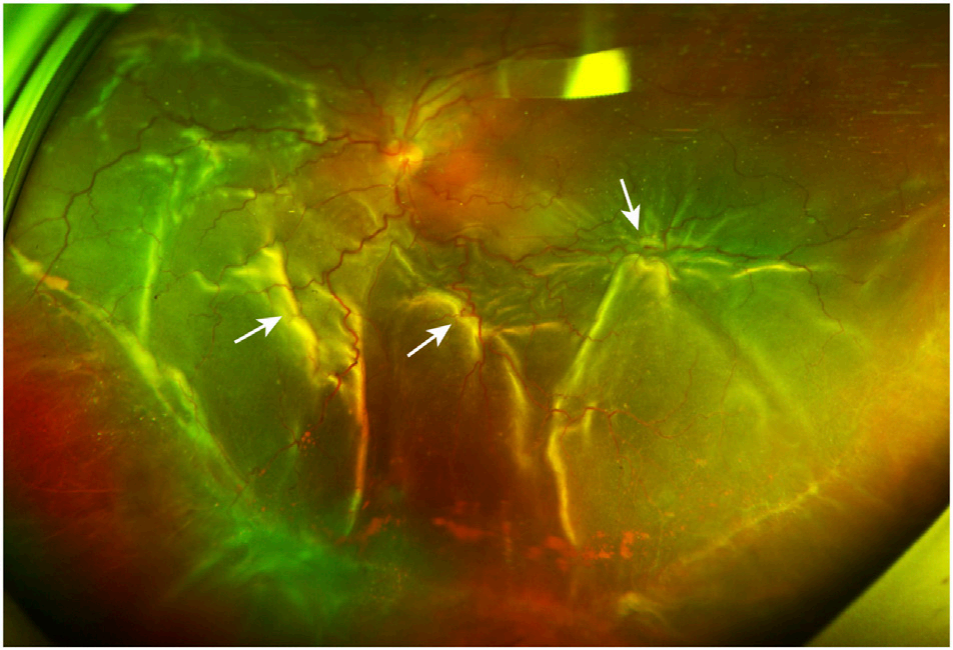
A PVR patient with retinal detachment. The ultra-widefield retinal image shows inferior vitreous hemorrhage and extensive retinal folds (white arrows) caused by PVRMs. *PVR, proliferative vitreoretinopathy; PVRM, proliferative vitreoretinopathy membrane.*

Proliferative vitreoretinopathy (PVR) is the major cause of failure after rhegmatogenous retinal detachment (RD) surgery and is responsible for 5–10% of all RD cases (Machemer et al., 1991; de la Rua et al., 2008). PVR is generally characterized by postoperative growth and contraction of fibrocellular membranes within the vitreous cavity and on both retinal surfaces and, in many cases, a fibrotic process of the retina itself (Pastor et al., 2002). These periretinal membranes create tangential traction force on the retina, and the distorted retina develops into fixed retinal folds, causing vision-compromising RD (Figure 1). Therefore, identifying and following individuals at high risk of developing PVR after RD surgery for early intervention have great practical significance. SNPs are important to genetic susceptibility and can serve as risk factors for multifactorial diseases in humans. For example, a case–control study by Pastor-Idoate et al. (2013a); Pastor-Idoate et al. (2013b) showed that the Pro variant of the *p53* codon 72 polymorphism (rs1042522) and the *MDM2* SNP309 G allele are associated with a higher risk of PVR among European subjects undergoing RD surgery. Given that rs1042522 and *MDM2* T309G synergistically attenuate the proapoptotic function of p53 in direct and indirect ways, respectively, they produce an additional effect on the risk of PVR in carriers harboring both SNPs (Pastor-Idoate et al., 2013b). This finding highlights the association of *MDM2* T309G and individual genetic susceptibility to PVR.

**FIGURE 2:**
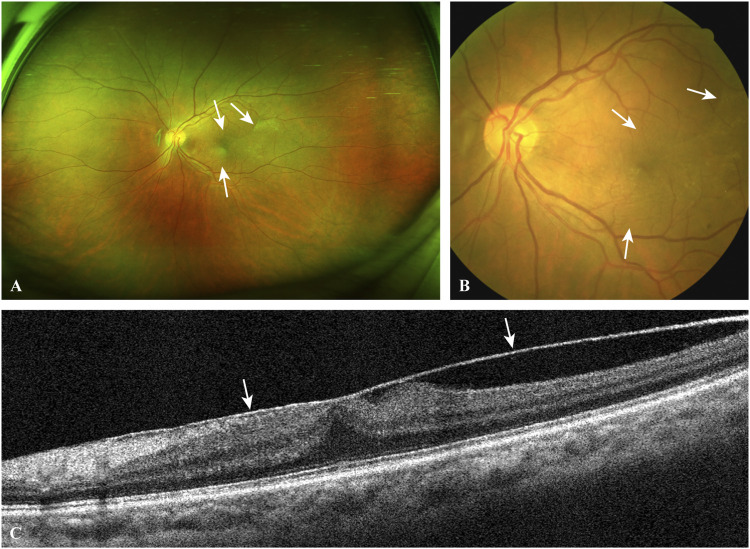
A typical case of EMM. The ultra-widefield retinal image **(A)** and the fundus photograph **(B)** show macular puckers (white arrows) caused by contracted EMMs. OCT scan **(C)** through the central fovea of **(A,B)** demonstrates a contracted EMM (white arrows) causing significant macular wrinkling and thickening. *EMM, epimacular membrane; OCT, optical coherence tomography.*

Epimacular membranes (EMMs) are relatively common sight-threatening conditions featuring fibrocellular proliferation along the surface of the internal limiting membrane (ILM) of the retina. These membranes preferentially grow in the macular region. Primary/idiopathic EMMs are strongly associated with the development of posterior vitreous detachment (PVD) and vitreomacular traction induced by age-related changes in the extracellular matrix (ECM) of the vitreoretinal interface (Bu et al., 2014). Other EMMs are secondary to various predisposing conditions, including diabetic retinopathy, retinal vein occlusion, rhegmatogenous RD, high myopia, ocular trauma, vitreous hemorrhage, retinal photocoagulation or cryotherapy, uveitis and cataract surgery. In the initial stage of EMM development, which is termed cellophane maculopathy, thin, translucent membranes increase the foveal light reflex under ophthalmoscopy, although patients are usually asymptomatic. In the progressive stage, known as macular puckers or preretinal macular fibrosis, the membranes become thickened and semitranslucent with incremental degrees of inflammation and fibrosis. Contractile EMMs generate retinal wrinkling and distort the macula, causing macular edema, ischemia or even macular holes (Figure 2). Typical manifestations are decreased visual acuity, central scotoma, metamorphopsia, micropsia/macropsia and monocular diplopia. According to previous histological studies, cellular components in EMMs include hyalocytes, retinal pigment epithelium (RPE) cells, Müller cells (MCs), astrocytes, microglia, macrophages, fibroblasts and myofibroblasts (Smiddy et al., 1989; Zhao et al., 2013; Vishwakarma et al. (2020) demonstrated that retinal glial cells are activated under oxidative stress and interact with other cells to cause neuroinflammation and neurodegeneration, which underlie the formation of EMMs under different pathological conditions. As the activation of oxidative stress and proinflammatory signaling as well as fibrocellular proliferation are, to some extent, modulated by the MDM2-p53 axis in these cells, we sought to determine whether *MDM2* T309G is related to EMMs. To the best of our knowledge, no studies have yet examined this issue. In this study, we aimed to investigate the role of *MDM2* T309G in EMM formation by comparing the genotype distribution and consistency of the polymorphism in paired membrane-blood samples between the PVR, EMM and healthy Chinese donor groups.

## Materials and Methods

### Design and Study Population

This was a cross–sectional study of patients who underwent pars plana vitrectomy for PVR or EMMs at the Second Xiangya Hospital of Central South University between 2020 and 2021. PVR membranes (PVRMs), ILMs removed along with PVRMs (PVR-ILMs) and peripheral venous blood samples from the same patients (PVR-blood) were collected from patients with PVR, and EMMs, EMM-ILMs and EMM-blood were collected from patients with EMMs. Membranes that were too small in size or compound membranes consisting of fibrocellular membranes and ILMs were first excluded.

For the PVR group, the inclusion criteria were an age older than 18 years, a diagnosis of rhegmatogenous RD by preoperative ocular examination (slit-lamp microscopy, optical coherence tomography (OCT), B-scan, fundus photography), and Machemer grade C1 PVR (Machemer et al., 1991) or higher, and the exclusion criterion was traumatic, tractional, exudative or iatrogenic RD. For the EMM group, the inclusion criteria were an age older than 18 years, Gass grade 2 EMMs diagnosed by preoperative ocular examination, complaint of decreased visual acuity, metamorphopsia or other visual defects, and the exclusion criterion was RD of any kind.

**FIGURE 3:**
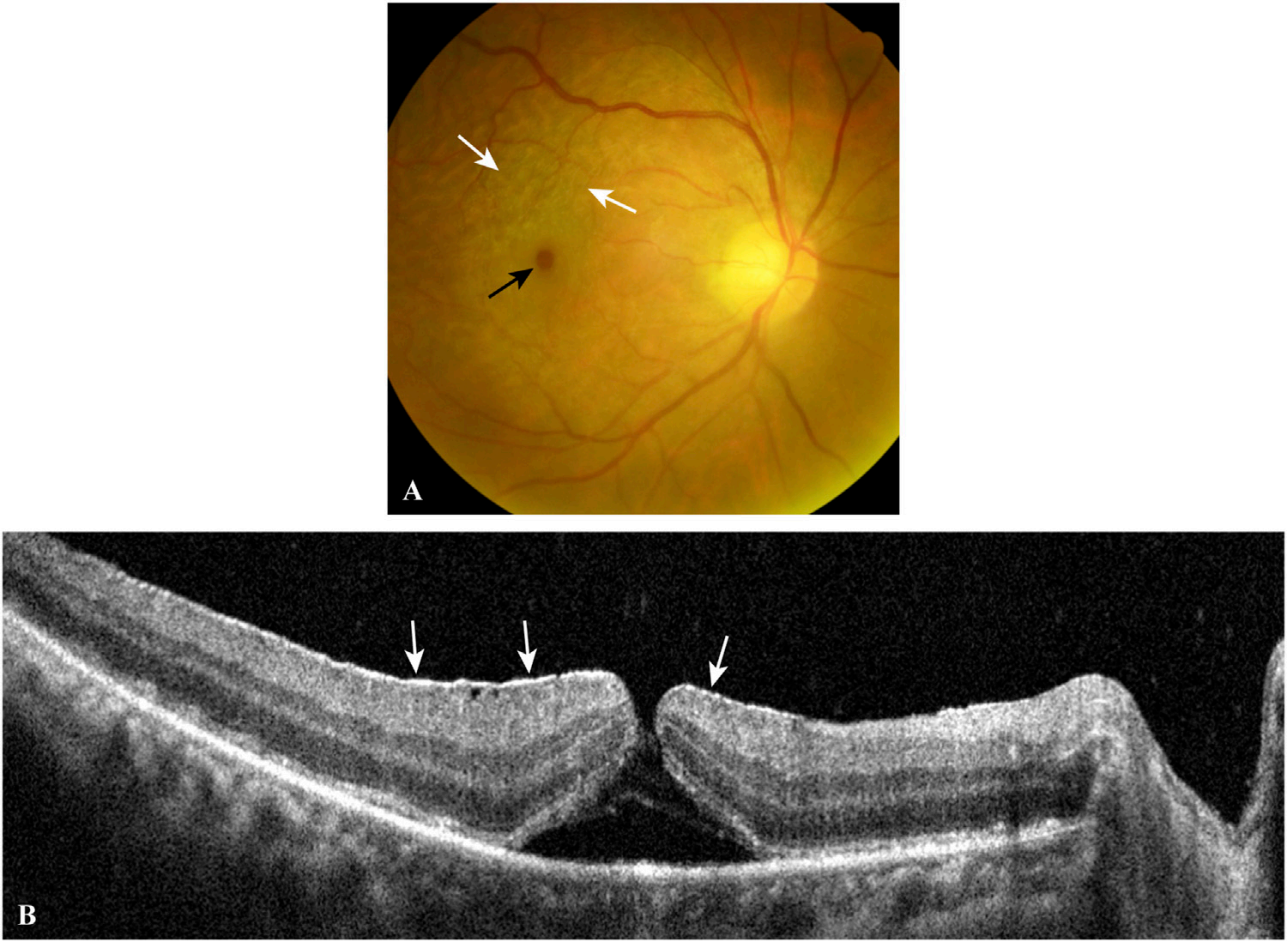
A typical case of EMM complicated by a macular hole. The fundus photograph **(A)** shows a macular hole (black arrow) and surrounding macular puckers (white arrows) caused by contracted EMMs. OCT scan **(B)** through the central fovea of **(A)** demonstrates a contracted EMM (white arrows) causing a macular hole with significant macular wrinkling and thickening. *EMM, epimacular membrane; OCT, optical coherence tomography.*

To investigate the association between macular holes and genomic stability at the *MDM2* T309G locus in EMMs and EMM-ILMs, we subdivided the EMM group into a macular hole subgroup and a control subgroup based on preoperative ocular examination. A patient belonged to the macular hole subgroup if signs of macular holes were found (Figure 3). Those without any signs of preoperative macular holes were included in the control subgroup.

### Genotyping

Genomic DNA was isolated from 300 μl of peripheral blood using the Wizard^®^ Genomic DNA Purification Kit (Promega, Madison, WI, United States) following the standard protocol. Membranes were ground by a KZ-II High-Speed Tissue Homogenizer (Servicebio, Wuhan, Hubei, PRC) in a clean, sterilized 1.8-ml tube with 150 μl of QuickExtract™ DNA Extraction Solution (Lucigen, Madison, WI, United States). The time and frequency were usually 120 s and 70 Hz, respectively, but varied from case to case. The homogenate was transferred to a 200-μl polymerase chain reaction (PCR) tube and boiled at 99°C for 40 min in a T100™ Thermal Cycler (Bio–Rad, Hercules, CA, United States). After centrifugation at 13,000 ×g for 5 min, the supernatant was retained and contained the genomic DNA of membrane cells.

The genomic DNA was subjected to PCR with the forward primer 5′-GGC​ACG​TGG​CTT​TGC​GGA​GG-3′ and reverse primer 5′-GCC​CCA​GCT​GGA​GAC​AAG​TC-3′ to obtain a 359-base pair DNA fragment. The 30-μl reaction mixture was composed of 2 μl of genomic DNA template, 2 μl of each primer at 10 μM, 9 μl of ddH_2_O and 15 μl of 2× Taq PCR Master Mix with Dye (Biosune, Shanghai, PRC; the mix contains dNTPs at 200 μM, MgCl_2_ at 1.5 mM and 0.5–0.7 U of Taq DNA polymerase). The PCR steps were an initial 4-min denaturation step at 95°C, followed by 35 cycles of 95°C for 40 s, 58°C for 40 s, 72°C for 40 s and a final elongation step at 72°C for 5 min. The amplified DNA fragments were separated on a 1.58% agarose gel and purified with a Gel/PCR DNA Purification Kit (Biosune, Shanghai, PRC) for bidirectional Sanger DNA sequencing performed by Shanghai Biosune Biotechnology Co., Ltd.

**FIGURE 4:**
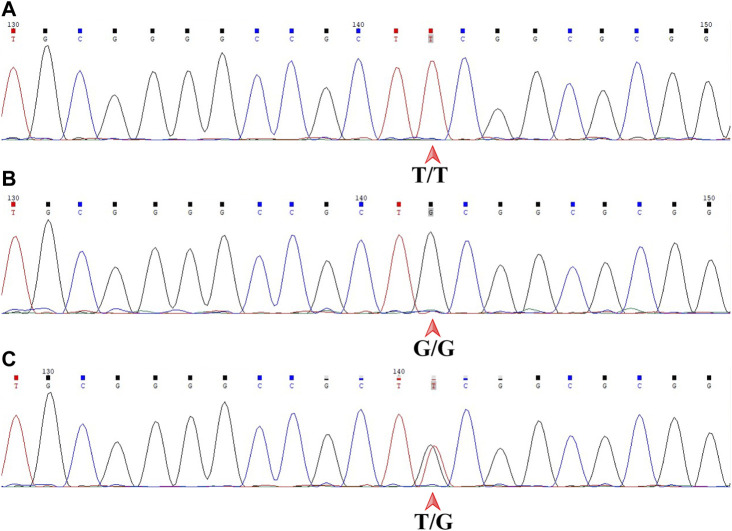
Sanger DNA sequencing of the 359-base pair DNA fragment in a forward read. *MDM2* SNP309 is downstream of 5′-CCGCT-3'. **(A)** T/T shown by a single red peak. **(B)** G/G shown by a single black peak. **(C)** T/G shown by overlapping red and black peaks. *MDM2, mouse double minute 2 gene; SNP, single-nucleotide polymorphism.*

The genotype of each sample was independently determined by two senior experts in a blinded fashion. Chromas software (version 2.6.6, Technelysium, Brisbane, QLD, AUS) was used to display the sequencing files. *MDM2* T309G is just downstream of 5′-CCGCT-3′ by forward sequencing and just upstream of 5′-AGCGG-3′ by reverse sequencing. The genotype of a sample was T/T, G/G or T/G, as indicated by a single red peak, a single black peak or overlapping red and black peaks by forward sequencing (Figure 4), which corresponded to A/A with a single green peak, C/C with a single blue peak or A/C with overlapping green and blue peaks by reverse sequencing. All samples included gave consistent results by forward and reverse sequencing.

### Statistical Analysis

Statistical analyses were conducted using GraphPad Prism software (version 9.0.0.121, GraphPad, San Diego, CA, United States). The sex composition and mean age of the study population were analyzed by the chi-square and unpaired t tests. The genotype distribution of *MDM2* T309G and the frequency of the *MDM2* SNP309 G allele in all samples were compared by the chi-square test. The somatic mutation rate at the *MDM2* T309G locus in all membrane samples was compared by the chi-square and Fisher’s exact tests, as in some cases, one or more theoretical frequencies were less than five, or the total number was less than 40. Two-sided *p* values with a significance level of 0.05 were obtained for all comparisons. Odds ratios (ORs) and 95% confidence intervals (95% CIs) were calculated using the Baptista-Pike and Wilson/Brown methods, respectively.

## Results

### Study Characteristics

According to diagnosis, the study population was divided into a PVR group and an EMM group. The final analysis included 123 subjects, consisting of 62 patients with PVR and 61 with EMMs. Table 1 summarizes the basic characteristics of all subjects. The two groups were statistically similar in sex composition based on the chi-square test (*p* = 0.1754). The mean age significantly differed between the two groups, as revealed by the unpaired *t* test (*p* < 0.0001). The EMM group (60.90 years, 95% CI, 58.13–63.68 years) was older on average than the PVR group (52.03 years, 95% CI, 48.73–55.33 years).

**TABLE 1:** Basic characteristics of the study population.

Characteristic	PVR group	EMM group
Sex		
Male, N	34	26
Female, N	28	35
Total, N	62	61
*p* Value	0.1754 (ns)	
Age		
Mean ± SEM (years)	52.03 ± 1.65	60.90 ± 1.39
95% CI	48.73–55.33	58.13–63.68
*p* Value	<0.0001 (****)	

95% CI, 95% confidence interval; EMM, epimacular membrane; ns, not significant; PVR, proliferative vitreoretinopathy; SEM, standard error of mean. Asterisks indicate statistical significance upon comparison: ****p < 0.0001.

### Genotype Distribution and Allelic Frequency of *MDM2* T309G

All samples were classified into three categories, each of which was composed of subsamples from the PVR group and the EMM group: fibrocellular membranes (PVRMs or EMMs), ILMs peeled together with fibrocellular membranes during surgery (PVR-ILMs or EMM-ILMs) and peripheral venous blood samples. The distribution of *MDM2* T309G in blood from healthy Chinese donors was derived from Knappskog et al. (2011). Fibrocellular membranes, ILMs and blood samples from both groups were genotyped by Sanger DNA sequencing. The statistics for the genotype distribution of *MDM2* T309G and the frequency of the *MDM2* SNP309 G allele (% G) are presented with details in Table 2. The proportion of the G allele number to the total allele number in each subsample is represented by % G, whereby a higher percentage indicates a greater impact of the G allele on disease pathogenesis. The genotype distribution was not significantly different within the PVR group (PVRMs, PVR-ILMs and PVR-blood; *p* = 0.9585) or EMM group (EMMs, EMM-ILMs and EMM-blood; *p* = 0.1996) (Table 2).

**TABLE 2:** Genotype Distribution of *MDM2* T309G and frequency of the *MDM2* SNP309 G allele in all samples.

	PVR group			EMM group			Healthy donors^1^
	PVRM	ILM	Blood	EMM	ILM	Blood	Blood
Genotype							
G/G, N	12	4	14	10	3	18	66
T/G, N	31	8	26	35	19	31	159
T/T, N	9	2	7	8	2	10	94
*p* Value	0.9585 (ns)			0.1996 (ns)			
T/G + G/G, N			40			49	225
T/T, N			7			10	94
*p* Value (compared to donors)			0.0369 (*)			0.0479 (*)	
OR			2.387			2.047	
95% CI			1.065–5.740			1.027–4.149	
Frequency of the G allele							
G allele, N			54			67	291
T allele, N			40			51	347
*p* Value (compared to donors)			0.0319 (*)			0.0256 (*)	
OR			1.610			1.567	
95% CI			1.034–2.499			1.046–2.335	
% G^2^			57.45%			56.78%	45.61%
95% CI			47.35–66.96%			47.77–65.36%	41.78–49.49%

95% CI, 95% confidence interval; EMM, epimacular membrane; ILM, internal limiting membrane; MDM2, mouse double minute 2 gene; ns, not significant; OR, odds ratio; PVR, proliferative vitreoretinopathy; PVRM, proliferative vitreoretinopathy membrane; SNP, single-nucleotide polymorphism. Asterisks indicate statistical significance upon comparison: *p < 0.05.1. The distribution of MDM2 T309G in blood from healthy Chinese donors was derived from Knappskog et al. (2011) 2. % G is the frequency of the MDM2 SNP309 G allele calculated by G allele N/total allele N×100%.

**FIGURE 5:**
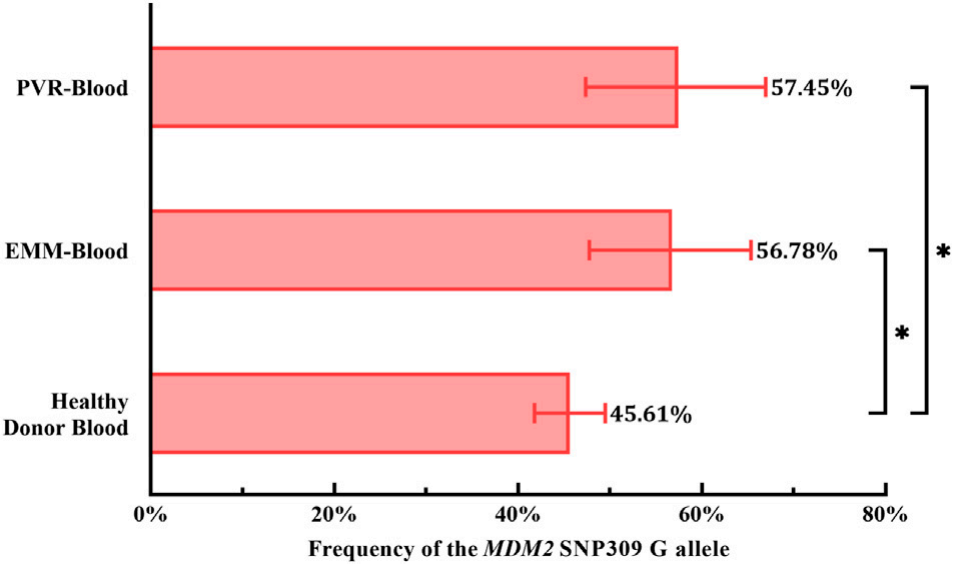
Comparison of the frequency of the *MDM2* SNP309 G allele in blood samples. Overall, both cohorts of patients exhibited a higher frequency of the G allele than healthy Chinese donors. *95% CI, 95% confidence interval; EMM, epimacular membrane; MDM2, mouse double minute 2 gene; PVR, proliferative vitreoretinopathy; SNP, single nucleotide polymorphism.* Asterisks indicate statistical significance upon comparison: *p < 0.05. Data are presented as the frequency of the *MDM2* SNP309 G allele ±95% CI.

To evaluate the role of the G allele in the pathogenesis of PVR and EMMs, we used the genotype distribution of *MDM2* T309G in blood from healthy Chinese donors in a study by Knappskog et al. (2011) because the genotype distribution varies by race. Based on chi-square tests, significant differences in the proportion of *MDM2* SNP309 G allele carriers (T/G + G/G) were observed between PVR-blood (*p* = 0.0369; OR = 2.387, 95% CI, 1.065–5.740) or EMM-blood (*p* = 0.0479; OR = 2.047, 95% CI, 1.027–4.149) and blood from healthy Chinese donors (Table 2). Additionally, the % G of PVR-blood (% G = 57.45%, 95% CI, 47.35–66.96%; *p* = 0.0319; OR = 1.610, 95% CI, 1.034–2.499) and EMM-blood (% G = 56.78%, 95% CI, 47.77–65.36%; *p* = 0.0256; OR = 1.567, 95% CI, 1.046–2.335) was significantly higher than that of blood from healthy donors (% G = 45.61%, 95% CI, 41.78–49.49%) (Figure 5; Table 2). Thus, the *MDM2* SNP309 G allele is associated with higher risks of both PVR and EMMs.

### Somatic Mutation at the *MDM2* T309G Locus

We found that the genotypes of the membranes were not always consistent with those of the matched blood samples. These membranes were considered somatically mutated. Genotype determined by Sanger DNA sequencing varied between mutated membranes and matched blood samples. In contrast, the results for unmutated membranes were consistent with those for matched blood samples. All pairs of mutated samples were examined *de novo* twice to exclude errors. The somatic mutation rate at the *MDM2* T309G locus (% M) was obtained by dividing the mutated membrane number by the paired membrane number times 100%.

**FIGURE 6:**
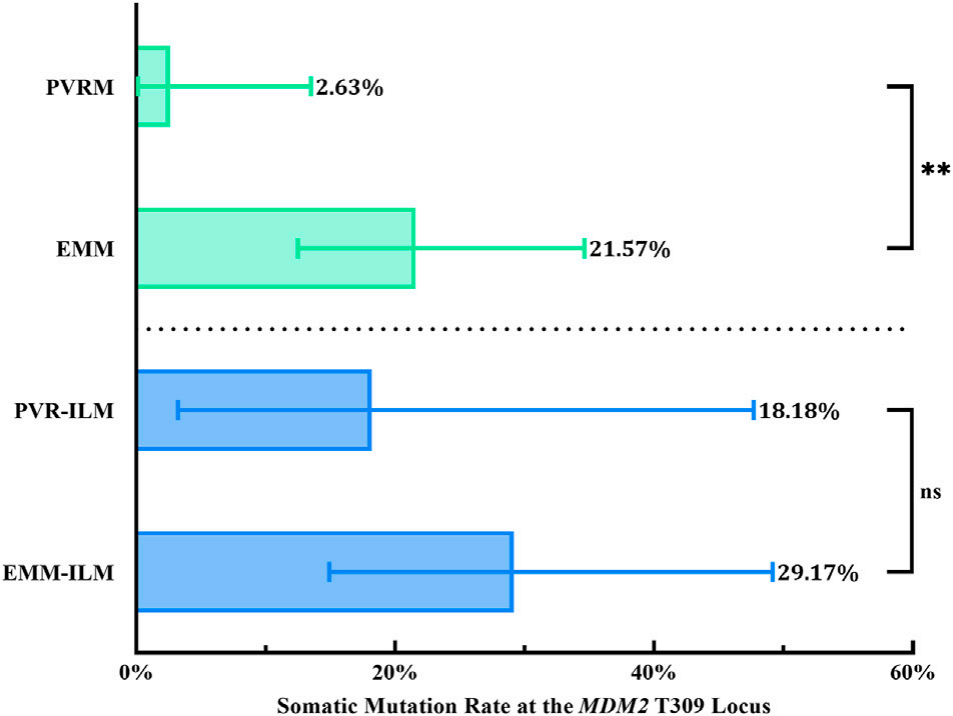
Somatic mutation at the *MDM2* T309G locus in membranes. EMMs were prone toward somatic mutation at the *MDM2* T309G locus, in contrast to PVRMs. PVR-ILMs and EMM-ILMs showed comparable mutation rates. *95% CI, 95% confidence interval; EMM, epimacular membrane; ILM, internal limiting membrane; MDM2, mouse double minute 2 gene; ns, not significant; PVRM, proliferative vitreoretinopathy membrane.* Asterisks indicate statistical significance upon comparison: **p < 0.01. Data are presented as the somatic mutation rate at the *MDM2* T309G locus ±95% CI.

Interestingly, the chi-square test revealed a significantly higher % M for EMMs than for PVRMs (% M of PVRMs = 2.63%, 95% CI, 0.13–13.49%; % M of EMMs = 21.57%, 95% CI, 12.49–34.63%; *p* = 0.0097; OR = 10.18, 95% CI, 1.594–112.4) (Figure 6; Table 3). To our surprise, ILMs in both groups showed a considerable % M by Fisher’s exact test (% M of PVR-ILMs: 18.18%, 95% CI, 3.23–47.70%; % M of EMM-ILMs: 29.17%, 95% CI, 14.91–49.17%; *p* = 0.6855; OR = 1.853, 95% CI, 0.3435–10.12) (Figure 6; Table 3). These results suggest that in the ILMs from both sources and EMMs, but not in the PVRMs, proliferating cells tended to incur somatic mutation at the *MDM2* T309G locus.

**TABLE 3:** Somatic mutation at the *MDM2* T309G locus in all membrane samples.

	Fibrocellular membrane		ILM	
	PVRM	EMM	PVR-ILM	EMM-ILM
Somatic mutation in membranes				
Mutated Membrane, N	1	11	2	7
Unmutated Membrane, N	37	40	9	17
*p* Value	0.0097 (**)		0.6855 (ns)	
OR	10.18		1.853	
95% CI	1.594–112.4		0.3435–10.12	
% M^1^	2.63%	21.57%	18.18%	29.17%
95% CI	0.13–13.49%	12.49–34.63%	3.23–47.70%	14.91–49.17%

95% CI, 95% confidence interval; EMM, epimacular membrane; ILM, internal limiting membrane; MDM2, mouse double minute 2 gene; ns, not significant; OR, odds ratio; PVR, proliferative vitreoretinopathy; PVRM, proliferative vitreoretinopathy membrane. Asterisks indicate statistical significance upon comparison: **p < 0.01.1. % M is the somatic mutation rate at the MDM2 T309G locus in a subset of membranes.

**FIGURE 7:**
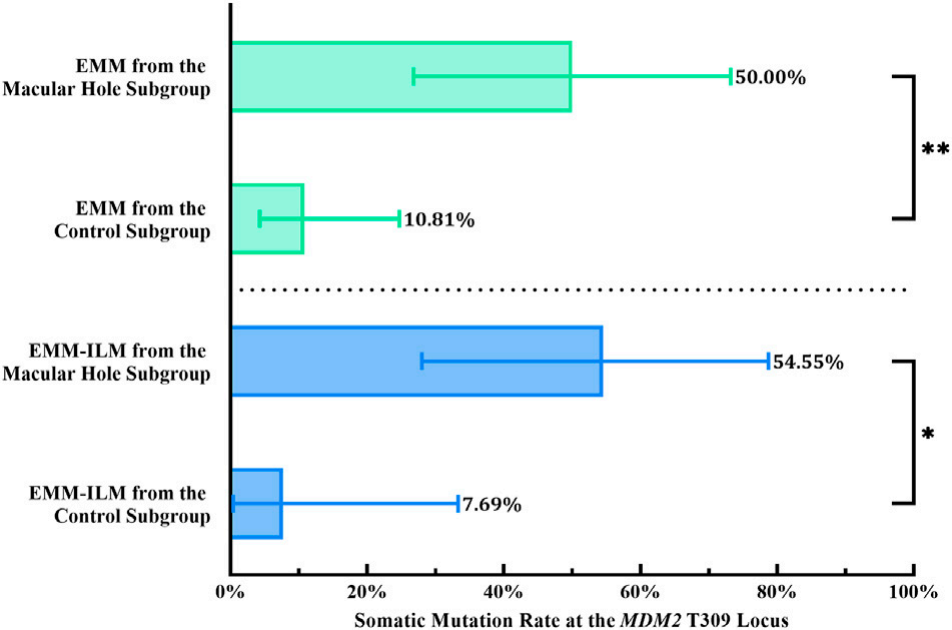
EMMs and ILMs from EMM cases with preoperative macular holes tended toward somatic mutation at the *MDM2* T309G locus. *EMM, epimacular membrane; ILM, internal limiting membrane; MDM2, mouse double minute 2 gene.* Asterisks indicate statistical significance upon comparison: *p < 0.05, **p < 0.01. Data are presented as the somatic mutation rate at the *MDM2* T309G locus ±95% CI.

Mechanical retinal damage, disruption of the outer blood–retinal barrier and exposure of the vitreous body are crucial steps in PVR pathogenesis. To investigate whether these factors are involved in somatic mutation at the *MDM2* T309G locus in EMMs and EMM-ILMs, the EMM group was subdivided into a macular hole subgroup and a control subgroup according to preoperative ocular examination. The % M of EMMs was higher for the macular hole subgroup than for the control subgroup, as demonstrated by Fisher’s exact test (% M of the macular hole subgroup = 50.00%, 95% CI, 26.80–73.20%; % M of the control subgroup: 10.81%, 95% CI, 4.29–24.71%; *p* = 0.0053; OR = 8.250, 95% CI, 1.668–29.20) (Figure 7; Table 4). Furthermore, EMM-ILMs from the macular hole subgroup were more likely to be mutated than EMM-ILMs from the control subgroup (% M of the macular hole subgroup: 54.55%, 95% CI, 28.01–78.73%; % M of the control subgroup: 7.69%, 95% CI, 0.39–33.31%; *p* = 0.0233; OR = 14.40, 95% CI, 1.560–175.8) (Figure 7; Table 4). In brief, EMMs and ILMs from EMM cases with preoperative macular holes tended to exhibit somatic mutation at the *MDM2* T309G locus.

**TABLE 4:** Association of somatic mutation at the *MDM2* T309G locus and macular holes in EMM cases.

	EMM		EMM-ILM	
	Macular hole subgroup	Control subgroup	Macular hole subgroup	Control subgroup
Somatic mutation in membranes				
Mutated Membrane, N	7	4	6	1
Unmutated Membrane, N	7	33	5	12
*p* Value	0.0053 (**)		0.0233 (*)	
OR	8.250		14.40	
95% CI	1.668–29.20		1.560–175.8	
% M^1^	50.00%	10.81%	54.55%	7.69%
95% CI	26.80–73.20%	4.29–24.71%	28.01–78.73%	0.39–33.31%

95% CI, 95% confidence interval; EMM, epimacular membrane; ILM, internal limiting membrane; MDM2, mouse double minute 2 gene; OR, odds ratio. Asterisks indicate statistical significance upon comparison: *p < 0.05, **p < 0.01.1. % M is the somatic mutation rate at the MDM2 T309G locus in a subset of membranes.

## Discussion

In the present study, we investigated the genotype distribution of *MDM2* T309G in fibrocellular membranes, ILMs and blood samples from PVR and EMM patients. For both groups, the proportion of *MDM2* SNP309 G allele carriers and the frequency of the G allele (% G) in blood samples were significantly higher than those in blood samples from the healthy donor group. Furthermore, between-group comparisons of *MDM2* T309G consistency in paired membrane-blood samples revealed that EMMs are predisposed toward somatic mutation at the *MDM2* T309G locus but that PVRMs are not, in contrast to PVR-ILMs and EMM-ILMs, which undergo mutation at similar ratios. Another notable finding was that EMMs and ILMs from EMM patients with preoperative macular holes had a greater mutation rate (% M) than those from patients without preoperative macular holes. Hence, we propose for the first time that *MDM2* T309G is associated with the development of EMMs in the Chinese population. That is, carriers of the *MDM2* SNP309 G allele are at a higher risk for developing EMMs. Moreover, EMMs and ILMs, especially those complicated with macular holes, are genetically unstable at the *MDM2* T309G locus. We hypothesize that MCs, which participate in reactive gliosis against various retinal stresses, or retinal progenitor/stem cells derived from dedifferentiated MCs are susceptible to oxygen free radical-induced single-base substitutions at the *MDM2* T309G locus. These cells may be responsible for the genomic instability in EMMs and ILMs, although this hypothesis needs to be further confirmed.

Our results are consistent with a previous report showing that age is a risk factor for EMMs but do not support female sex as a risk factor (Xiao et al., 2017). The average age of the EMM group was 8.87 years older than that of the PVR group, possibly due to the rapid worsening of visual function in PVR patients or the prolonged development of EMMs. Nevertheless, this study is not a large-scale epidemiological survey, and the above results are for reference only. We believe that the difference in average age is mainly attributed to the intrinsic properties of the two diseases and hardly affects our results, as such a difference is far from enough to have a statistically significant impact on overall genomic stability.

PVR is a pathological vitreoretinal fibrosis condition induced by vitreal growth factors, cytokines and inflammatory mediators produced by RPE cells, MCs, microglial cells and macrophages, including platelet-derived growth factor (PDGF), TGF-β2, EGF, FGF2, and TNF-α. Stimulated by these vitreal factors, detached RPE cells undergo epithelial to mesenchymal transition (EMT), acquiring enhanced proliferation and migration capacities, resistance to apoptosis and the ability to produce ECM (Chiba, 2014). The transformed cells then migrate into the subretinal space or, through retinal breaks, the vitreous cavity. They proliferate and transdifferentiate into fibroblasts and myofibroblasts together with other cells to form subretinal or epiretinal PVRMs (Agrawal et al., 2007; Pastor et al., 2016). Therefore, researchers employ PVR vitreous from patients or animals to induce EMT in RPE cells for experiments. In recent years, multiple studies have confirmed that MDM2 is involved in PVR. Initially, chronic activation of Akt and suppression of p53 were identified by Lei et al. (2011) as a signature pathway downstream of the PDGF receptor α (PDGFR-α) signaling cascade in PVR. However, stabilizing intracellular p53 levels by Nutlin-3, a small-molecule antagonist of the MDM2-p53 interaction, abrogated the Akt-p53 pathway and prevented human PVR vitreous-induced contraction of cells isolated from a PVRM (Lei et al., 2012). In fact, PVR vitreous induces ERK/Sp1-dependent MDM2 upregulation and subsequent p53 downregulation in RPE cells. The resultant enhanced abilities of RPE cells to proliferate, survive, contract and migrate are intrinsic to PVR. Inspired by previous works by Pastor-Idoate et al. (2013b), Lei’s team introduced *MDM2* T309G into RPE cells to create RPE cells with *MDM2* SNP309 T/T and *MDM2* SNP309 T/G. Importantly, PVR vitreous-induced cellular responses were enhanced in T/G RPE cells compared with T/T RPE cells, but this was abrogated by genetic silencing of aberrant P2-driven expression of MDM2 caused by *MDM2* T309G (Duan et al., 2016; Zhou et al., 2017; Chen et al., 2019). *MDM2* T309G also promoted the development of experimental PVR in an *in vivo* rabbit model (Zhou et al., 2017). Our results show a larger proportion of G allele carriers in the PVR group than in healthy donors and a higher % G in blood samples from PVR patients than from healthy donors. This finding is consistent with the established notion that *MDM2* T309G promotes PVR. It has been proposed that aberrant P2-driven inducible expression of MDM2 facilitates EMT in RPE cells by targeting E-cadherin, a cell adhesion protein and a key regulator of EMT, among others, for ubiquitin-dependent proteasomal degradation (Yang et al., 2006; Liu et al., 2019). Thus, carriers of the *MDM2* SNP309 G allele exhibit a greater increase in MDM2 levels in response to RD-induced stress. Such an overabundance of MDM2 induces RPE cells to undergo EMT, thereby contributing to PVRM formation. Most importantly, we show that G allele carriers were at a higher risk of developing EMMs than non-G allele carriers (*p* = 0.0479; OR = 2.047, 95% CI, 1.027–4.149) and that EMM patients exhibited a significantly higher % G than healthy donors (*p* = 0.0256; OR = 1.567, 95% CI, 1.046–2.335), indicating the role of the *MDM2* SNP309 G allele in promoting EMMs (Figure 5; Table 2).

Surprisingly, of all 124 pairs of matched membrane-blood samples, 21 pairs were inconsistent in genotype. Moreover, possible operational errors were excluded by duplicate examination of both forward and reverse sequencing. Since peripheral blood leukocytes are typically genetically stable, such inconsistency arises from somatic mutations in membrane cells under pathological conditions. A likely explanation for this finding is that mutation is a consequence of overproduced reactive oxygen species in the vitreal inflammatory response. Reactive oxygen species are constantly produced through normal cellular oxygen respiration or stress responses to various environmental mutagens and carcinogens, such as ionizing radiation, chemicals and ultraviolet light. Excessive oxygen free radicals attack DNA and its precursor deoxyribonucleotides, leading to DNA damage and mutations. The most studied oxidative DNA lesion, 8-oxo-7,8-dihydro-2′-deoxyguanosine (8-oxo-dG)/8-hydroxy-2′-deoxyguanosine (8-OH-dG, an enol tautomer of 8-oxo-dG), has been strongly implicated in cancers due to its capacity to induce the G/C → T/A transversion (Moriya, 1993; Valko et al., 2006). Furthermore, free nucleotides are oxidized more efficiently than DNA, and oxidized dNTPs cause potent genomic instability when they are incorporated into daughter strands during DNA replication (Maki, 2002). 8-Hydroxy-2′-deoxyguanosine 5′-triphosphate (8-OH-dGTP, an equivalent of 8-oxo-dGTP) and 2-hydroxy-2′-deoxyadenosine 5′-triphosphate (2-OH-dATP, an equivalent of 2-oxo-dATP), the oxidized forms of dGTP and dATP, respectively, induce A/T → C/G and G/C → T/A transversions, respectively, in newly synthesized DNA (Inoue et al., 1998). These single-base substitutions are elicited by human DNA polymerase η-, ζ- or REV1-mediated misincorporation of 8-OH-dGTP opposite template adenine and 2-OH-dATP opposite template guanine (Maki and Sekiguchi, 1992; Inoue et al., 1998; Hidaka et al., 2008; Satou et al., 2009). Correspondingly, of the 21 membrane samples with heterozygous mutations in this study, 6 and 15 harbored an A/T → C/G transversion and a G/C → T/A transversion at the *MDM2* T309G locus, respectively.

An interesting finding is that % M varied between PVRMs and EMMs (*p* = 0.0097; OR = 10.18, 95% CI, 1.594–112.4) but not between PVR-ILMs and EMM-ILMs (*p* = 0.6855; OR = 1.853, 95% CI, 0.3435–10.12), suggesting that EMMs and ILMs are intrinsically connected in terms of genomic instability at the *MDM2* T309G locus (Figure 6; Table 3). Histologically, Müller glial cells are a major cellular component of EMMs (Zhao et al., 2013). These cells are a major type of macroglia that maintain retinal homeostasis and provide structural, metabolic and trophic support. Their radial processes extend across the entire neurosensory retina, serving as living optical fibers. Above all, MCs also play a pivotal role in retinal gliosis. Reactive MC gliosis is a protective process against retinal injury that insulates the retina from vitreal pathogenic factors, promotes retinal tissue regeneration and limits tissue remodeling (Bringmann and Wiedemann, 2012). Under diverse pathological conditions, MCs in the mature retina rapidly proliferate and dedifferentiate to pluripotent retinal progenitor/stem cells, which migrate along MC processes to the damaged retinal layer and redifferentiate to replenish lost neurons (Reichenbach and Bringmann, 2013). Any retinal or vitreal insult might trigger this reparative reaction, and common initiating factors are RD, retinal ischemia, PVD, vitreous hemorrhage, endophthalmitis and trauma. However, excessive gliosis leads to glial scars, which are aberrant tissue repairs in response to retinal stress. In particular, various EMMs (primary and secondary) are a distinct type of glial scar attached to the retina by hypertrophied MC fibers (Bringmann and Wiedemann, 2012). In response to stress, retinal glial cells, notably MCs, migrate to the vitreoretinal interface through an intact or defective ILM, proliferating and transdifferentiating, along with hyalocytes, RPE cells, macrophages, fibroblasts and myofibroblasts, on the scaffold of the ILM (collagen IV) to form early EMMs. In brief, EMMs essentially constitute progressive fibrocellular proliferation on the vitreal side of the ILM and are inextricably linked to the ILM. As routine clinical procedures, vitrectomy and the removal of EMMs along with ILM peeling clear the hazardous local microenvironment and relieve macular traction, thus favoring postoperative anatomic and visual recovery of the retina. The ILM is the innermost layer of the neurosensory retina that adheres the vitreous to the retina. It is a thin basement membrane formed by MC endfeet. ECM proteins that make up the ILM, including collagen IV, laminins, nidogens and heparan sulfate proteoglycans, are believed to be produced and secreted by MCs (Peynshaert et al., 2019; Zhang and Johnson, 2021). Based on the above facts, it is reasonable to speculate that MCs are responsible for the propensity of EMMs and ILMs toward somatic mutation at the *MDM2* T309G locus.

Last but not least, the heightened % M observed in EMMs (*p* = 0.0053; OR = 8.250, 95% CI, 1.668–29.20) and ILMs (*p* = 0.0233; OR = 14.40, 95% CI, 1.560–175.8) from EMM patients with preoperative macular holes compared with those without holes (Figure 7; Table 4) further corroborates this speculation, as oxidative stress and aberrant preretinal gliosis intensify with retinal injury, giving rise to genomic instability at the *MDM2* T309G locus in highly proliferating MCs. We hypothesize that the blood–retinal barrier adjacent to macular holes is disrupted and that leukocytes emigrate to the preretinal space or vitreous cavity, releasing cytokines and inflammatory mediators. Inflammatory infiltration produces vast amounts of oxygen free radicals that attack proliferating MCs in EMMs and ILMs and cause somatic mutation at the *MDM2* T309G locus. Admittedly, further research is needed to confirm our conjecture.

The major limitations of this study include the following: 1) An insufficient sample size. A substantial number of compound membranes consisting of fibrocellular membranes and ILMs and their matched blood samples were unqualified for this study and excluded from the cohorts. The association between *MDM2* T309G and EMMs may need to be further validated with a larger sample size. 2) A lack of blood samples from healthy donors. The genotype distribution of *MDM2* T309G varies with race (Wilkening et al., 2007; Knappskog et al., 2011). Therefore, the statistics of genotype distribution for healthy Chinese donors (Knappskog et al., 2011) were selected for comparison to minimize racial/ethnic bias. 3) This study was conducted in only the Chinese population, and it is unclear whether these conclusions can be extended to other ethnicities. Hence, replication studies in larger independent cohorts of different ethnicities are required.

In summary, this study highlights the role of the *MDM2* SNP T309G in the development of EMMs. Herein, we report for the first time that the *MDM2* SNP309 G allele is an associated factor for EMMs in a Chinese population. This observation provides new insights into the molecular mechanism of these pathologies, which may be beneficial for developing novel approaches and enabling early intervention for high-risk populations to improve visual prognosis.

## Data Availability

The original contributions presented in the study are included in the article/Supplementary Material, further inquiries can be directed to the corresponding authors.

## Abbreviations

95% CI, 95% confidence interval; 2-OH-dATP, 2-hydroxy-2′-deoxyadenosine 5′-triphosphate; 8-OH-dGTP, 8-hydroxy-2′-deoxyguanosine 5′-triphosphate; ECM, extracellular matrix; EMM, epimacular membrane; EMT, epithelial to mesenchymal transition; ILM, internal limiting membrane; MDM2, mouse double minute 2 gene; MC, Müller cell; ns, not significant; OCT, optical coherence tomography; OR, odds ratio; PCR, polymerase chain reaction; PDGFR-α, platelet-derived growth factor receptor α; PVD, posterior vitreous detachment; PVR, proliferative vitreoretinopathy; PVRM, proliferative vitreoretinopathy membrane; RPE, retinal pigment epithelium; RD, retinal detachment; SEM, standard error of mean; SNP, single-nucleotide polymorphism.
